# Bis(ethyl­enedithio)tetra­thia­fulvalenium–tetra­chloridocobaltate(II) (3/2)

**DOI:** 10.1107/S1600536808033692

**Published:** 2008-11-08

**Authors:** Fuqi Zhao, Ping Li, Xiaohui Zhu, Lihua Dong

**Affiliations:** a School of Chemistry and Chemical Engineering, TaiShan Medical University, Tai’an 271016, People’s Republic of China

## Abstract

The structure of the electrochemically crystallized title compound, (C_10_H_8_S_8_)_3_[CoCl_4_]_2_, consists of two types of bis­(ethyl­enedithio)tetra­thia­fulvalene (BEDT-TTF) radical cation stacks separated by sheets of tetra­hedral [CoCl_4_]^2−^ anions. One of the BEDT-TTF mol­ecules is generated by inversion. There are short S⋯S contacts between the stacks in the *a* direction and short C—H⋯Cl contacts between the radical cations and the anions.

## Related literature

For related literature, see: Mori (1998 ▶, 1999 ▶); Mori *et al.* (1999 ▶, 2002 ▶); Shibaeva & Yagubskii (2004 ▶); Varma *et al.* (1987 ▶); Williams *et al.* (1984 ▶).
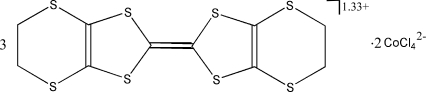


## Experimental

### 

#### Crystal data

(C_10_H_8_S_8_)_3_[CoCl_4_]_2_
*M* *_r_* = 1555.39
Triclinic, 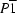

*a* = 6.7904 (19) Å
*b* = 9.6402 (18) Å
*c* = 20.499 (4) Å
α = 86.280 (14)°
β = 89.967 (19)°
γ = 78.456 (19)°
*V* = 1311.9 (5) Å^3^
*Z* = 1
Mo *K*α radiation
μ = 2.03 mm^−1^
*T* = 293 (2) K
0.22 × 0.18 × 0.10 mm

#### Data collection

Bruker P4 diffractometer
Absorption correction: ψ scan (*XSCANS*; Bruker, 1996 ▶) *T*_min_ = 0.652, *T*_max_ = 0.816
6527 measured reflections
5159 independent reflections
1927 reflections with *I* > 2σ(*I*)
*R*_int_ = 0.094
97 standard reflections every 3 reflections intensity decay: 1%

#### Refinement

*R*[*F* ^2^ > 2σ(*F* ^2^)] = 0.100
*wR*(*F* ^2^) = 0.281
*S* = 0.92
5159 reflections
289 parameters
8 restraints
H-atom parameters constrained
Δρ_max_ = 0.91 e Å^−3^
Δρ_min_ = −1.06 e Å^−3^

### 

Data collection: *XSCANS* (Bruker, 1996 ▶); cell refinement: *XSCANS*; data reduction: *XSCANS*; program(s) used to solve structure: *SIR97* (Altomare *et al.*, 1999 ▶); program(s) used to refine structure: *SHELXL97* (Sheldrick, 2008 ▶); molecular graphics: *SHELXTL* (Sheldrick, 2008 ▶); software used to prepare material for publication: *SHELXTL*.

## Supplementary Material

Crystal structure: contains datablocks global, I. DOI: 10.1107/S1600536808033692/hb2784sup1.cif

Structure factors: contains datablocks I. DOI: 10.1107/S1600536808033692/hb2784Isup2.hkl

Additional supplementary materials:  crystallographic information; 3D view; checkCIF report

## Figures and Tables

**Table 1:** Selected bond lengths (Å)

Co1—Cl1	2.296 (5)
Co1—Cl2	2.265 (5)
Co1—Cl3	2.259 (5)
Co1—Cl4	2.276 (5)

**Table 2:** Hydrogen-bond geometry (Å, °)

*D*—H⋯*A*	*D*—H	H⋯*A*	*D*⋯*A*	*D*—H⋯*A*
C1—H1*A*⋯Cl1^i^	0.97	2.55	3.494 (16)	165
C1—H1*B*⋯Cl2^ii^	0.97	2.83	3.591 (16)	136
C10—H10*A*⋯Cl4^iii^	0.97	2.78	3.57 (2)	139

Symmetry codes: (i) 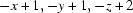; (ii) 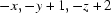; (iii) 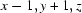.

**Fig. 1.**
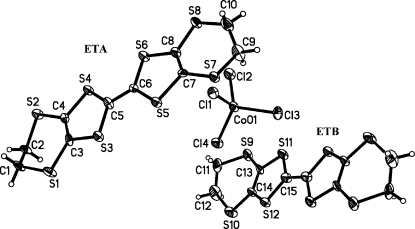
Molecular structure of (I) with displacement ellipsoids for the non-hydrogen atoms at the 30% probability level. The unlabelled atoms of the ETB molecule are generated by the symmetry operation (1-x, 1-y, 1-x).

**Fig. 2.**
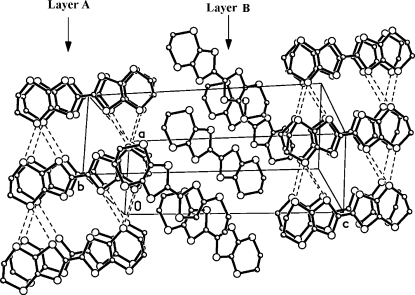
A packing diagram of (I), with short S···S contacts indicated by dashed lines. The anions and H atoms are omitted for clarity.

## supplementary crystallographic information

### Comment

There is a large variety of quasi-two-dimensional organic conductors based on the radical cation salts of bis(ethylenedithio)tetrathiafulvalene (BEDT-TTF) which also is abbreviated to ET. They show a wealth of structural modifications, but the conducting properties of the radical salts are essentially determined by the packing patterns of their BEDT-TTF layers (Shibaeva & Yagubskii, 2004). So, according to packing patterns of the BEDT-TTF layers in the various crystal structure, it is customary classified as α, β, δ, χ and so on. (Mori, 1998; Mori, 1999; Mori *et al.*, 1999)

In the title structure, (I), the unit cell contains three ET donors and two anions. Since one BEDT-TTF molecule (ETA) and [CoCl_4_]^2-^ anion are on general positions, and the other BEDT-TTF molecule (ETB) is positioned on an inversion center, the crystallographically independent molecules are 1.5 ET molecules and one [CoCl_4_]^2-^ anion (Fig. 1). The [CoCl_4_]^2-^ anion shows a slightly distorted tetrahedral configuration (Table 1). The two independent ET molecules have different molecular configuration. In the ETA, the terminal ethylene C2 and C9 atoms extend from the molecular plane with deviations of -0.499 (16) Å and -0.579 (18) Å, but C1 and C10 atoms [with deviations of 0.169 (17) Å and 0.057 (19) Å] almost share the same plane with the rest of the molecule. ETA shows a boat conformation in which C2 and C9 are on the both sides of the molecular plane. Otherwise, ETB shows a chair conformation in which C11 and C12 deviate from the molecular plane by 0.658 (18) Å and 0.02 (2) Å respectively and the inversion center positioned on the middle of the C15—C15A bond. The dihedral angle between the molecular planes of ETA and ETB is 16.88 (16)° and their molecular planes make angles of 43.93 (10)° and 27.10 (13)° with [010] directions respectively.

ETA and ETB form stack A and stack B along the *b* axis in a face to face manner with different intra-stack packing modes. In the stack A, ETA molecules are dimerized and the intra-stack packing mode alternates between the ROB [ring over bond, ET molecules stack on top of each other with a relative displacement between stacking moleculaes along their long in-plane axes] and ROA [ring over atom, ET molecules stack on above or below with a large displacement betweentacking moleculaes along their short in-plane axes] modes, as defined by Williams *et al.* (1984). The mean interplannar distance in the dimersied ET molecules is 3.606 Å and between the dimerized is 3.082 Å. There are three kinds of intra-dimer S···S short distances within each dimmer shorter than the combined van der Walls radii of two sulfur atoms (3.60 Å), but there are no obvious inter-dimer S···S short contacts between the dimers. Stack B contains only ETB molecules overlapped with ROB mode, and there are no intrastack S···S short contacts. The mean interplannar distance in stack B is 4.392 Å.

Stack A and Stack B form layer A and layer B in the *ab* plane respectively and these layers are interleaved by the inorganic anions. In layer A, one ET molecule has four pairs of inter-stack S···S short contacts between the stacks in the a-direction [S1···S2 = 3.518 (6) Å, S1···S4 = 3.562 (6) Å, S7···S8 = 3.549 (6) Å] as shown in Fig. 2. Combined with intra-stack S···S short contacts within Stack A, it is suggested that the possibility of a quasi-two-dimensional interaction in the *ab*-plane. In contrast to the layer A, there are no S···S distance are shorter than the sum of van der Walls radii of two sulfur atoms in the layer B, which indicates the absence of any intermolecular interaction in the layer B.

Between layer A and layer B, there are layers of [CoCl_4_]^2-^ anions, which engage in ionic donor-anion interactions.  The anions sandwich the layers A and layers B along the *c* axis. The [CoCl_4_]^2-^ anions are situated in the so called 'anion cavity', which is bonded by the terminal ethylene groups of the ET cations.  Short C—H···Cl interactions (Table 2) probably stabilize the crystal structure.

Among the molecular conductors based on the ET, there are three major determinant of packing of a crystal: (1) the ET network packing motif; (2) the anion layer motif; and (3) the interatomic environment at the interface of the donor and anion layers. For the larger size of the ET and fractional charge on it, the coulomb interaction between the ET cations and anions is inferior to the π-π interaction. In the title crystal, the ET molecules form poly-ET cations by the π-π interaction and [CoCl_4_]^2-^ anions fill the cavity formed by the ethylene groups of the surrounding ET molecules to balance charge. Hence, the 'anion cavity' is mainly determined by the ET packing motif where the 'anion cavity' is a trigonal channel along the [100] directions. If the size of anion is not enough to fill the anion cavity, the solvent molecules will be crystallized in the crystal to stabilize the structure, such as TCE(1,1,2-trichloroethane) molecules in the crystal α-ET_3_(CoCl_4_)(TCE)(Mori *et al.*,  2002). In the crystal α-ET_3_(CoCl_4_)(TCE), the oxidation on ET molecules is lower than that in the title compound, where only one [CoCl_4_]^2-^ anion is needed to balance charge, so the TCE solvent molecules are filled in the 'anion cavity' together. At the same time, if some of the anions are replaced by the other anion with same geometry, size and charge, the crystal structure will be an isomorphic structure, for example, β-(ET)_3_(CoCl_4_)_1.34_(GaCl_4_)_0.66_, α-ET_3_(CoCl_4_)_0.38_(CoCl_4_)_0.62_(TCE) (Mori *et al.*, 2002). On the other hand, the oxidation on ET molecules and the interaction between the anions and the ethylene groups of the ET will significant affect the packing motif of the ET cations, so the ET packing motif of the title crystal belongs to β' and ET_3_(CoCl_4_)(TCE) belongs to α. But the 'anion cavity' formed by the ET cations as above two packing motif are nearly same.

### Experimental

BEDT-TTF was synthesized following the method of Varma *et al.* (1987) and recrystallized from CHCl_3_. K_2_CoCl_4_.6H_2_O was synthesized by metathesis of CoCl_2_.6H_2_O and KCl in a molar ratio of 1:2 in water and recrystallized before use. All solvent were distilled before use.

Electrochemical crystal growth was carried out in conventional H-shaped cells with Pt electrodes in a constant temperature of 295 (2) K at a current of 0.2 µA. Each cell contained 7 mg of BEDT-TTF and 99.3 mg of K_2_CoCl_4_.6H_2_O together with 40 mg of 18-crown-6 in 35 mL of TCE(1,1,2-trichloroethane) under an inert atmosphere (N_2_).  Black needles of (I) were obtained after three months.

### Refinement

The electrocrystallization process is a 'self-assembly' process which is difficult to control. The tendency to grow as bundles of fine needles has hampered our efforts to get a better crystal. The low quality of the crystal was the reason for low bond precision, but the obtained structure information of the title compound is meaningful.

The H atoms were geometrically placed (C—H = 0.97Å) and refined as riding with *U*_iso_(H)= 1.2 *U*_eq_(C).

### Crystal data

**Table:**

(C_10_H_8_S_8_)_3_[CoCl_4_]_2_	*Z* = 1
*M**_r_* = 1555.39	*F*_000_ = 778
Triclinic, *P*1	*D*_x_ = 1.969 Mg m^−^^3^
Hall symbol: -P 1	Mo *K*α radiation λ = 0.71073 Å
*a* = 6.7904 (19) Å	Cell parameters from 31 reflections
*b* = 9.6402 (18) Å	θ = 5.0–12.4º
*c* = 20.499 (4) Å	µ = 2.03 mm^−^^1^
α = 86.280 (14)º	*T* = 293 (2) K
β = 89.967 (19)º	Prism, black
γ = 78.456 (19)º	0.22 × 0.18 × 0.10 mm
*V* = 1311.9 (5) Å^3^	

### Data collection

**Table:**

Bruker P4 diffractometer	*R*_int_ = 0.094
Radiation source: fine-focus sealed tube	θ_max_ = 26.0º
Monochromator: graphite	θ_min_ = 2.0º
*T* = 293(2) K	*h* = −8→1
/w scans	*k* = −11→11
Absorption correction: ψ scan(XSCANS; Bruker, 1996)	*l* = −25→25
*T*_min_ = 0.652, *T*_max_ = 0.816	97 standard reflections
6527 measured reflections	every 3 reflections
5159 independent reflections	intensity decay: 1%
1927 reflections with *I* > 2σ(*I*)	

### Refinement

**Table:**

Refinement on *F*^2^	Secondary atom site location: difference Fourier map
Least-squares matrix: full	Hydrogen site location: inferred from neighbouring sites
*R*[*F*^2^ > 2σ(*F*^2^)] = 0.100	H-atom parameters constrained
*wR*(*F*^2^) = 0.281	*w* = 1/[σ^2^(*F*_o_^2^) + (0.1192*P*)^2^] where *P* = (*F*_o_^2^ + 2*F*_c_^2^)/3
*S* = 0.92	(Δ/σ)_max_ < 0.001
5159 reflections	Δρ_max_ = 0.91 e Å^−^^3^
289 parameters	Δρ_min_ = −1.06 e Å^−^^3^
8 restraints	Extinction correction: none
Primary atom site location: structure-invariant direct methods	

### Special details

**Table:**

Geometry. All e.s.d.'s (except the e.s.d. in the dihedral angle between two l.s. planes) are estimated using the full covariance matrix. The cell e.s.d.'s are taken into account individually in the estimation of e.s.d.'s in distances, angles and torsion angles; correlations between e.s.d.'s in cell parameters are only used when they are defined by crystal symmetry. An approximate (isotropic) treatment of cell e.s.d.'s is used for estimating e.s.d.'s involving l.s. planes.
Refinement. Refinement of *F*^2^ against ALL reflections. The weighted *R*-factor *wR* and goodness of fit *S* are based on *F*^2^, conventional *R*-factors *R* are based on *F*, with *F* set to zero for negative *F*^2^. The threshold expression of *F*^2^ > σ(*F*^2^) is used only for calculating *R*-factors(gt) *etc*. and is not relevant to the choice of reflections for refinement. *R*-factors based on *F*^2^ are statistically about twice as large as those based on *F*, and *R*- factors based on ALL data will be even larger.

### Fractional atomic coordinates and isotropic or equivalent isotropic displacement parameters (Å^2^)

**Table:**

	*x*	*y*	*z*	*U*_iso_*/*U*_eq_	
C11	0.271 (3)	0.0230 (19)	0.6376 (9)	0.068 (6)	
H11A	0.2624	0.0986	0.6671	0.081*	
H11B	0.1845	−0.0397	0.6542	0.081*	
C12	0.487 (2)	−0.0600 (19)	0.6370 (11)	0.085 (7)	
H12A	0.4917	−0.1379	0.6090	0.102*	
H12B	0.5226	−0.1013	0.6809	0.102*	
C1	0.557 (2)	0.4107 (18)	1.1811 (8)	0.051 (5)	
H1A	0.6758	0.3413	1.1952	0.061*	
H1B	0.5116	0.4665	1.2180	0.061*	
C2	0.397 (2)	0.3341 (16)	1.1632 (8)	0.041 (4)	
H2A	0.3816	0.2680	1.1997	0.049*	
H2B	0.4443	0.2784	1.1265	0.049*	
C3	0.397 (2)	0.5999 (16)	1.0767 (7)	0.035 (4)	
C4	0.218 (2)	0.5659 (13)	1.0853 (7)	0.029 (3)	
C5	0.161 (2)	0.7692 (15)	0.9978 (7)	0.035 (4)	
C6	0.076 (2)	0.8723 (13)	0.9480 (7)	0.023 (3)	
C7	0.023 (2)	1.0697 (16)	0.8548 (7)	0.035 (4)	
C8	−0.155 (2)	1.0292 (14)	0.8635 (7)	0.034 (4)	
C9	−0.106 (3)	1.2069 (19)	0.7415 (9)	0.061 (6)	
H9A	−0.0977	1.2849	0.7099	0.073*	
H9B	−0.0827	1.1209	0.7181	0.073*	
C10	−0.304 (3)	1.228 (2)	0.7659 (9)	0.069 (6)	
H10A	−0.3953	1.2454	0.7286	0.082*	
H10B	−0.3260	1.3151	0.7888	0.082*	
C13	0.350 (2)	0.2161 (15)	0.5480 (7)	0.035 (4)	
C14	0.542 (2)	0.1863 (16)	0.5711 (8)	0.040 (4)	
C15	0.485 (3)	0.4319 (15)	0.5147 (9)	0.045 (4)	
Cl1	0.0026 (7)	0.7913 (4)	0.7470 (2)	0.0460 (11)	
Cl2	−0.2946 (7)	0.5703 (5)	0.6666 (2)	0.0563 (13)	
Cl3	0.0831 (8)	0.7338 (5)	0.5751 (2)	0.0546 (13)	
Cl4	0.2378 (7)	0.4170 (4)	0.6910 (2)	0.0529 (12)	
Co01	0.0140 (4)	0.6252 (2)	0.67099 (11)	0.0402 (6)	
S1	0.6258 (6)	0.5265 (5)	1.1162 (2)	0.0508 (13)	
S2	0.1496 (6)	0.4391 (4)	1.1417 (2)	0.0426 (11)	
S3	0.4092 (6)	0.7397 (4)	1.0199 (2)	0.0373 (10)	
S4	0.0217 (6)	0.6592 (4)	1.0356 (2)	0.0351 (10)	
S5	0.2191 (6)	0.9787 (4)	0.9081 (2)	0.0371 (10)	
S6	−0.1704 (6)	0.9004 (4)	0.9241 (2)	0.0370 (10)	
S7	0.0938 (6)	1.1928 (4)	0.7998 (2)	0.0388 (11)	
S8	−0.3763 (6)	1.0934 (5)	0.8193 (2)	0.0439 (11)	
S9	0.1832 (8)	0.0971 (5)	0.5591 (2)	0.0572 (14)	
S10	0.6771 (8)	0.0341 (5)	0.6107 (3)	0.0673 (16)	
S11	0.2669 (7)	0.3772 (5)	0.5071 (2)	0.0536 (13)	
S12	0.6655 (6)	0.3251 (4)	0.5600 (2)	0.0450 (12)	

### Atomic displacement parameters (Å^2^)

**Table:**

	*U*^11^	*U*^22^	*U*^33^	*U*^12^	*U*^13^	*U*^23^
C11	0.089 (17)	0.062 (13)	0.058 (13)	−0.042 (13)	0.003 (12)	0.028 (10)
C12	0.104 (18)	0.063 (13)	0.098 (17)	−0.053 (14)	−0.022 (14)	0.035 (12)
C1	0.038 (10)	0.046 (10)	0.060 (12)	0.004 (9)	−0.018 (9)	0.028 (9)
C2	0.028 (9)	0.030 (9)	0.060 (11)	−0.002 (7)	−0.011 (8)	0.018 (8)
C3	0.047 (10)	0.037 (9)	0.025 (8)	−0.024 (8)	−0.011 (8)	0.012 (7)
C4	0.039 (9)	0.014 (7)	0.031 (8)	−0.006 (7)	−0.001 (7)	0.011 (6)
C5	0.039 (10)	0.029 (8)	0.042 (9)	−0.023 (8)	−0.015 (8)	0.014 (7)
C6	0.024 (8)	0.017 (7)	0.028 (8)	−0.005 (6)	−0.003 (7)	−0.007 (6)
C7	0.027 (9)	0.032 (8)	0.040 (10)	0.007 (7)	0.007 (8)	0.008 (7)
C8	0.040 (10)	0.018 (7)	0.044 (10)	−0.008 (7)	−0.001 (8)	0.010 (7)
C9	0.065 (13)	0.054 (12)	0.074 (13)	−0.048 (11)	−0.022 (11)	0.025 (10)
C10	0.040 (12)	0.090 (16)	0.074 (14)	−0.023 (12)	−0.020 (11)	0.035 (12)
C13	0.041 (10)	0.029 (8)	0.036 (9)	−0.010 (8)	−0.013 (8)	0.013 (7)
C14	0.031 (9)	0.030 (8)	0.060 (11)	−0.016 (7)	−0.013 (8)	0.026 (8)
C15	0.043 (10)	0.021 (8)	0.069 (12)	−0.009 (8)	0.000 (9)	0.011 (8)
Cl1	0.058 (3)	0.028 (2)	0.053 (3)	−0.013 (2)	−0.001 (2)	0.0053 (18)
Cl2	0.053 (3)	0.047 (3)	0.071 (3)	−0.020 (2)	−0.004 (3)	0.013 (2)
Cl3	0.071 (3)	0.045 (3)	0.050 (3)	−0.021 (2)	0.003 (2)	0.013 (2)
Cl4	0.047 (3)	0.031 (2)	0.077 (3)	−0.006 (2)	−0.002 (2)	0.016 (2)
Co01	0.0444 (15)	0.0261 (11)	0.0492 (14)	−0.0093 (11)	−0.0038 (12)	0.0124 (10)
S1	0.030 (2)	0.044 (3)	0.076 (3)	−0.011 (2)	−0.013 (2)	0.028 (2)
S2	0.035 (2)	0.032 (2)	0.060 (3)	−0.014 (2)	−0.002 (2)	0.024 (2)
S3	0.032 (2)	0.038 (2)	0.044 (2)	−0.0170 (19)	−0.005 (2)	0.0166 (18)
S4	0.030 (2)	0.027 (2)	0.048 (3)	−0.0129 (18)	−0.0060 (19)	0.0179 (17)
S5	0.030 (2)	0.031 (2)	0.050 (3)	−0.0120 (18)	−0.004 (2)	0.0195 (18)
S6	0.030 (2)	0.030 (2)	0.053 (3)	−0.0170 (18)	−0.007 (2)	0.0143 (18)
S7	0.033 (2)	0.035 (2)	0.047 (3)	−0.011 (2)	−0.002 (2)	0.0167 (19)
S8	0.034 (2)	0.043 (2)	0.053 (3)	−0.012 (2)	−0.010 (2)	0.019 (2)
S9	0.060 (3)	0.052 (3)	0.065 (3)	−0.034 (3)	−0.014 (3)	0.030 (2)
S10	0.058 (3)	0.030 (2)	0.105 (4)	0.003 (2)	−0.008 (3)	0.030 (3)
S11	0.041 (3)	0.044 (3)	0.075 (3)	−0.019 (2)	−0.015 (2)	0.031 (2)
S12	0.035 (2)	0.038 (2)	0.062 (3)	−0.015 (2)	−0.009 (2)	0.018 (2)

### Geometric parameters (Å, °)

**Table:**

C11—C12	1.527 (10)	C7—C8	1.35 (2)
C11—S9	1.772 (17)	C7—S7	1.723 (14)
C11—H11A	0.9700	C7—S5	1.774 (15)
C11—H11B	0.9700	C8—S6	1.717 (14)
C12—S10	1.782 (9)	C8—S8	1.735 (16)
C12—H12A	0.9700	C9—C10	1.42 (2)
C12—H12B	0.9700	C9—S7	1.786 (17)
C1—C2	1.49 (2)	C9—H9A	0.9700
C1—S1	1.803 (16)	C9—H9B	0.9700
C1—H1A	0.9700	C10—S8	1.791 (17)
C1—H1B	0.9700	C10—H10A	0.9700
C2—S2	1.818 (15)	C10—H10B	0.9700
C2—H2A	0.9700	C13—C14	1.36 (2)
C2—H2B	0.9700	C13—S11	1.708 (14)
C3—C4	1.333 (19)	C13—S9	1.771 (15)
C3—S3	1.738 (14)	C14—S12	1.719 (14)
C3—S1	1.747 (16)	C14—S10	1.720 (15)
C4—S4	1.739 (14)	C15—C15^i^	1.46 (3)
C4—S2	1.756 (13)	C15—S12	1.672 (17)
C5—C6	1.416 (18)	C15—S11	1.679 (16)
C5—S3	1.708 (15)	Co1—Cl1	2.296 (5)
C5—S4	1.708 (13)	Co1—Cl2	2.265 (5)
C6—S6	1.706 (14)	Co1—Cl3	2.259 (5)
C6—S5	1.720 (13)	Co1—Cl4	2.276 (5)
			
C12—C11—S9	112.6 (14)	S6—C8—S8	115.0 (9)
C12—C11—H11A	109.1	C10—C9—S7	117.0 (15)
S9—C11—H11A	109.1	C10—C9—H9A	108.0
C12—C11—H11B	109.1	S7—C9—H9A	108.0
S9—C11—H11B	109.1	C10—C9—H9B	108.0
H11A—C11—H11B	107.8	S7—C9—H9B	108.0
C11—C12—S10	117.6 (12)	H9A—C9—H9B	107.3
C11—C12—H12A	107.9	C9—C10—S8	119.3 (14)
S10—C12—H12A	107.9	C9—C10—H10A	107.5
C11—C12—H12B	107.9	S8—C10—H10A	107.5
S10—C12—H12B	107.9	C9—C10—H10B	107.5
H12A—C12—H12B	107.2	S8—C10—H10B	107.5
C2—C1—S1	114.6 (12)	H10A—C10—H10B	107.0
C2—C1—H1A	108.6	C14—C13—S11	117.8 (11)
S1—C1—H1A	108.6	C14—C13—S9	123.2 (11)
C2—C1—H1B	108.6	S11—C13—S9	119.0 (9)
S1—C1—H1B	108.6	C13—C14—S12	113.9 (11)
H1A—C1—H1B	107.6	C13—C14—S10	130.2 (11)
C1—C2—S2	117.9 (11)	S12—C14—S10	115.8 (9)
C1—C2—H2A	107.8	C15^i^—C15—S12	121.2 (17)
S2—C2—H2A	107.8	C15^i^—C15—S11	122.1 (17)
C1—C2—H2B	107.8	S12—C15—S11	116.7 (8)
S2—C2—H2B	107.8	Cl3—Co01—Cl2	110.4 (2)
H2A—C2—H2B	107.2	Cl3—Co01—Cl4	110.28 (19)
C4—C3—S3	116.7 (12)	Cl2—Co01—Cl4	106.69 (18)
C4—C3—S1	129.2 (11)	Cl3—Co01—Cl1	105.49 (17)
S3—C3—S1	114.1 (8)	Cl2—Co01—Cl1	107.4 (2)
C3—C4—S4	116.9 (10)	Cl4—Co01—Cl1	116.55 (19)
C3—C4—S2	128.5 (12)	C3—S1—C1	103.6 (7)
S4—C4—S2	114.6 (8)	C4—S2—C2	99.8 (7)
C6—C5—S3	122.8 (10)	C5—S3—C3	95.1 (7)
C6—C5—S4	120.8 (11)	C5—S4—C4	95.0 (7)
S3—C5—S4	116.3 (8)	C6—S5—C7	95.2 (7)
C5—C6—S6	123.3 (10)	C6—S6—C8	96.0 (7)
C5—C6—S5	120.7 (11)	C7—S7—C9	98.3 (8)
S6—C6—S5	116.0 (8)	C8—S8—C10	101.3 (8)
C8—C7—S7	131.0 (12)	C13—S9—C11	97.4 (8)
C8—C7—S5	114.8 (11)	C14—S10—C12	103.2 (8)
S7—C7—S5	114.2 (9)	C15—S11—C13	94.8 (7)
C7—C8—S6	118.0 (12)	C15—S12—C14	96.2 (7)
C7—C8—S8	127.0 (11)		
			
S9—C11—C12—S10	61 (2)	C3—C4—S4—C5	−3.4 (14)
S1—C1—C2—S2	−63.6 (17)	S2—C4—S4—C5	175.8 (9)
S3—C3—C4—S4	3.4 (18)	C5—C6—S5—C7	178.4 (12)
S1—C3—C4—S4	−178.5 (10)	S6—C6—S5—C7	−2.0 (9)
S3—C3—C4—S2	−175.7 (9)	C8—C7—S5—C6	0.7 (14)
S1—C3—C4—S2	2(2)	S7—C7—S5—C6	−178.4 (9)
S3—C5—C6—S6	178.5 (9)	C5—C6—S6—C8	−178.0 (13)
S4—C5—C6—S6	2.7 (19)	S5—C6—S6—C8	2.4 (9)
S3—C5—C6—S5	−1.9 (18)	C7—C8—S6—C6	−2.0 (14)
S4—C5—C6—S5	−177.7 (8)	S8—C8—S6—C6	177.8 (9)
S7—C7—C8—S6	179.7 (10)	C8—C7—S7—C9	−18.6 (19)
S5—C7—C8—S6	0.9 (18)	S5—C7—S7—C9	160.3 (10)
S7—C7—C8—S8	0(3)	C10—C9—S7—C7	49.3 (16)
S5—C7—C8—S8	−178.9 (9)	C7—C8—S8—C10	−3.3 (18)
S7—C9—C10—S8	−64 (2)	S6—C8—S8—C10	176.9 (10)
S11—C13—C14—S12	4.6 (19)	C9—C10—S8—C8	35.6 (19)
S9—C13—C14—S12	−174.7 (9)	C14—C13—S9—C11	34.6 (17)
S11—C13—C14—S10	−176.7 (11)	S11—C13—S9—C11	−144.8 (10)
S9—C13—C14—S10	4(3)	C12—C11—S9—C13	−64.9 (14)
C4—C3—S1—C1	−8.6 (18)	C13—C14—S10—C12	−17 (2)
S3—C3—S1—C1	169.6 (9)	S12—C14—S10—C12	161.7 (11)
C2—C1—S1—C3	37.9 (15)	C11—C12—S10—C14	−17 (2)
C3—C4—S2—C2	−17.8 (17)	C15^i^—C15—S11—C13	177 (2)
S4—C4—S2—C2	163.1 (9)	S12—C15—S11—C13	−5.1 (12)
C1—C2—S2—C4	49.0 (14)	C14—C13—S11—C15	0.1 (15)
C6—C5—S3—C3	−176.9 (13)	S9—C13—S11—C15	179.5 (11)
S4—C5—S3—C3	−0.9 (11)	C15^i^—C15—S12—C14	−175 (2)
C4—C3—S3—C5	−1.4 (14)	S11—C15—S12—C14	7.2 (12)
S1—C3—S3—C5	−179.9 (9)	C13—C14—S12—C15	−6.9 (15)
C6—C5—S4—C4	178.4 (13)	S10—C14—S12—C15	174.3 (11)
S3—C5—S4—C4	2.3 (11)		

Symmetry codes: (i) −*x*+1, −*y*+1, −*z*+1.

### Hydrogen-bond geometry (Å, °)

**Table:**

*D*—H···*A*	*D*—H	H···*A*	*D*···*A*	*D*—H···*A*
C1—H1A···Cl1^ii^	0.97	2.55	3.494 (16)	165
C1—H1B···Cl2^iii^	0.97	2.83	3.591 (16)	136
C10—H10A···Cl4^iv^	0.97	2.78	3.57 (2)	139

Symmetry codes: (ii) −*x*+1, −*y*+1, −*z*+2; (iii) −*x*, −*y*+1, −*z*+2; (iv) *x*−1, *y*+1, *z*.
